# Barriers to Asthma Treatment in the United States: Results From the Global Asthma Physician and Patient Survey

**DOI:** 10.1097/WOX.0b013e3181c81ea4

**Published:** 2009-12-15

**Authors:** Michael S Blaiss, Michael A Kaliner, Carlos E Baena-Cagnani, Ronald Dahl, Erkka J Valovirta, Giorgio W Canonica

**Affiliations:** 1 University of Tennessee Health Science Center, Memphis, TN; 2 Institute for Asthma and Allergy, Chevy Chase, MD; 3 Centre of Respiratory Medicine and Allergy. Chutro Clinic Córdoba, Córdoba, Argentina; 4 Aarhus University Hospital, Department of Respiratory Diseases, Aarhus, Denmark; 5 Turku Allergy Center, Turku, Finland; 6 Allergy and Respiratory Diseases Clinic, University of Genova DIMI, Genova, Italy

**Keywords:** asthma, inhaled corticosteroids, side-effects, Global Asthma Physician and Patient (GAPP) Survey, compliance, asthma management, asthma education

## Abstract

**Background:**

The Global Asthma Physician and Patient (GAPP) survey evaluated the perceptions of both physicians and patients on the management of asthma. Here we present the results from the United States (US) subpopulation of the GAPP survey.

**Methods:**

The GAPP Survey was a large, global study (physicians, n = 1733; patients, n = 1726; interviews, n = 3459). In the US, 208 adults (aged ≥ 18 years) with asthma and 224 physicians were recruited. Respondents were questioned using self-administered online interviews with close-ended questionnaires.

**Results:**

Physician and patient responses were found to differ in regard to perception of time spent on asthma education, awareness of disease symptoms and their severity, asthma medication side effects, and adherence to treatment and the consequence of nonadherence. Comparison of the US findings with the global GAPP survey results suggest the US physician-patient partnership compared reasonably well with the other countries in the survey. Both patients and physicians cited a need for new asthma medication.

**Conclusions:**

Similar to the global GAPP survey, the US-specific findings indicate that in general there is a lack of asthma control, poor adherence to therapy, and room for improvement in patient-physician communication and partnership in treating asthma.

## 

Asthma is a major public health problem that affects an estimated 20 million people in the United States (US), with the prevalence expected to continue to rise in the future [1-3]. Asthma is characterized by airway inflammation and hyper-responsiveness that lead to symptoms such as coughing, wheezing, breathlessness and chest tightness [1]. The impact of asthma on patients can be considerable,[4] and the chronic characteristic of the disease may require long-term controller therapy to help manage symptoms and sustain lung function [1]. Inadequately controlled asthma can result in substantial absence from school or lost productivity at work, and a reduced quality of life [2,5-7].

Effective medications are available to help control the persistent symptoms of asthma and appropriate intervention with these is an essential part of any asthma management program [1]. Inhaled corticosteroids (ICS) are the most effective controller therapy currently available and are recommended by the international (GINA) and national (NAEPP/NHLBI) guidelines as the first-line treatment for persistent asthma in all patients [1,8]. However, utilization of these medications is suboptimal [9,10] and adherence can also be poor, which can limit the therapeutic outcomes [11].

The Global Asthma Physician and Patient (GAPP) Survey was the first global quantitative survey developed to obtain the opinions on the management and treatment of asthma from both physicians and patients [12]. This survey directly questioned physicians and patients from 16 countries on asthma management practices, treatment patterns, adherence to therapy, and opinions of current medication. Results from the global GAPP Survey indicated that there is strong relationship between a patient's adherence to therapy and the quality of physician-patient communication and the level of medication side-effects [12]. The global GAPP survey also found that although ICS are indicated by the guidelines as first-line therapy for asthma [1,8] physicians tend to under-prescribe ICS and may even prescribe long acting *β*-agonists (LABAs) for mild persistent asthma for which the GINA and NHLBI guidelines recommend ICS monotherapy [1,8,12]. Because of country-specific social and health care environments, country-specific findings may differ from the global GAPP results. This article presents the results from the US subpopulation of the global GAPP Survey.

## Methods

The GAPP Survey was a large, global research study conducted by Harris Interactive (Rochester, NY) between May 18 and August 24, 2005 in 16 countries (Australia, Belgium, Brazil, Canada, France, Germany, Ireland, Italy, Japan, The Netherlands, Poland, Spain, Switzerland, South Africa, the United Kingdom, and the US) on behalf of the GAPP Survey global advisory board, which was comprised of one member from the American College of Allergy, Asthma and Immunology and 5 members from the World Allergy Organization [12]. The survey interviewed adults (≥ 18 years of age) with asthma, generalists (ie, family practitioners, general practitioners, internists), and specialists (ie, allergists and pulmonologists). For more details regarding the global survey see Reference 12.

Sample size for the US was ~200 asthmatic adults and ~200 physicians. Patients with asthma were recruited by phone from the Harris Interactive Inc.'s Chronic Illness and Global Consumer Panels, in which several thousand patients are registered [13]. US physicians were recruited and surveyed through Harris Interactive Inc.'s Online Physician Panel; this panel includes every major medical specialty and is representative of the US physician population by region, sex, and medical specialty [13]. Individuals were randomly selected from these panels.

For this survey there was no specific approval or informed consent. However, to be a member of the Harris Interactive Inc.'s Chronic Illness and Global Consumer Panels or Harris Interactive Inc.'s Online Physician Panel required that participating patients and physicians consent that their responses can be used in public as long as they were presented in an aggregate, anonymous, and confidential fashion.

Physicians were included in the survey if they were currently practicing medicine and had been doing so for 3 to 30 years, saw at least 3 adult asthma patients per week and wrote at least one asthma medication prescription per week. Patients had to have diagnosed asthma and be ≥ 18 years of age, although, patients with chronic obstructive pulmonary disease were not excluded.

The questionnaires were developed by the GAPP survey global advisory board. Before use the questionnaire was tested on ten people in the US to ensure the content and language of the questions were generally understood. Physicians and patient respondents were questioned on topics pertinent to asthma treatment using layperson and medical language as appropriate and included physician prescribing habits and beliefs, patient's perception of their asthma, patient-doctor communication, and asthma medication issues. Questions regarding demographic information were also included to help further define and understand responses.

The number of interviews conducted was determined to ensure statistical significance could be measured globally and in each country for physicians and adult patients. Globally, a total of 3459 interviews were conducted with 1733 physicians and 1726 adult patients via the Internet, telephone, or in person [12]. Respondents from the US were questioned using self-administered online interviews with close-ended questionnaires that took an average of 20 minutes to complete. The full content of both questionnaires are provided as supplemental material (see Tables 1 and 2; **Supplemental Digital Content 1**, http://links.lww.com/WAOJOURNAL/A1).

**Table 1:** Physicians and Patients Demographics

Physicians, n	224
Speciality, n (%)	
Allergist	11 (5)
Pulmonologist	4 (2)
Family medicine	81 (36)
General practice	16 (7)
Internal medicine	112 (50)
Gender, n (%)	
Male	152
Mean amount of time in clinical practice, years	15.5
Practice profile	
Mostly office/clinic based, %	89
Mean number of patients/week, (%)	
1-60	13
61-100	43
> 100	45
Asthma experience	
Mean number of adult patients/week, n	22.2
Mean number of prescriptions/week, n	37.5
Patients with asthma	
Patients, n	208
Gender, n (%)	
Female	122 (59)
Mean age, years	43.4
Years since diagnosis, n (%)	
< 5	38 (18)
5 to < 10	38 (18)
10 to < 15	37 (18)
15 to < 20	35 (17)
20 to < 30	27 (13)
≥ 30	33 (16)

**Table 2:** Patient Satisfaction With Current Asthma Treatments

Property of Current Asthma Treatment	Patients Satisfied (%)
Ease of use	92
Effectiveness	89
Fast acting	85
Safety	84
Dosing frequency per day	84
Potential for side-effects	74

Patient question: Overall, how satisfied are you with the following features of your current asthma medication or medications*?

*Nonspecific with regard to asthma medication.

Physician data were weighted by specialty, sex, and years in practice to reflect the profile of physicians in the American Medical Association. Patient data were weighted by sex, education, age, household, income, and region to reflect the adult asthma patient population from the National Health Interview. With the number of physicians and patients being ~200 each, the sampling error for both groups was ± 3% and the margin of error was ± 7%.

## Results

In the US, 208 adults with asthma (mean age, 43.4 years) and 224 physicians (mean duration in practice, 15.5 years) responded to the questionnaire (Table 1). The rates of response for physicians was 30% and for adults with asthma 5%. The majority of participating physicians specialized in internal medicine (50%) or family medicine (36%) (Table 1). The mean number of adult asthma patients seen per week was 22.2, and the mean number of adult asthma prescriptions written per week was 37.5 (Table 1). The majority of patients were being treated by a primary care physician (64%) (Table 1). Fifteen percent were being treated by specialists (allergist or pulmonologist) and over one-fifth (21%) were not having their asthma treated by any physician or healthcare professional (Table 1; see **Supplemental Table **1, **Q1a, Supplemental Digital Content 1**, http://links.lww.com/WAOJOURNAL/A1).

### Impact of Asthma and Disease Control

Out of 208 adult patients interviewed 64% described their asthma as mild, 30% as moderate, and 6% as severe (Q2a). When asked whether asthma limited their daily activities, 38% of patients reported that it affected them 'not at all,' 31% responded 'not much,' 30% 'somewhat,' and 2% 'a great deal' (Q3a). Never-the-less, many of the patients may not have had well-controlled asthma, as 20% of respondents reported making an unscheduled telephone call to the doctor, 20% an unscheduled office visit, 6% an emergency department visit, and 4% reported being admitted to hospital in association with their asthma over the last year (Q4a).

### Current Treatment

**Figure 1:**
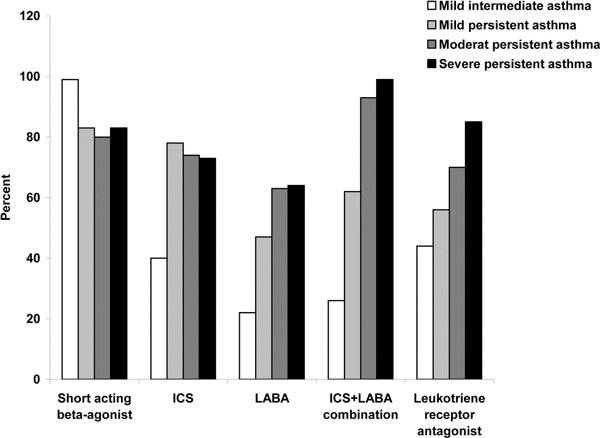
**Asthma medications currently being prescribed by physicians as first-line therapy for differing severities of asthma**. Physician question: Which medication or medications* do you prescribe as first-line treatment for: 1) mild intermittent asthma; 2) mild persistent asthma; 3) moderate persistent asthma; and 4) severe persistent asthma? *Physicians could provide more than one medication as a first-line treatment for each severity of asthma.

When patients were asked about which medications they were currently receiving for their asthma, combination therapy (an ICS plus a long-acting *β*_2_-agonist [LABA]), ICS monotherapy, and leukotriene receptor antagonists were reported as being the most commonly used (29%, 27%, and 25%, respectively), followed by LABAs (7%) and anticholinergics (5%) (Q5a). Of patients interviewed, 30% also reported receiving 'other' forms of medication (Q5a). For first-line treatment, most physicians reported prescribing short acting *β*-agonists for all asthma severities and an ICS plus LABA combination for moderate and severe persistent asthma (Figure 1; see **Supplemental Table **2, **Q1b, Supplemental Digital Content 1**, http://links.lww.com/WAOJOURNAL/A1). The majority of physicians reported prescribing multiple medications for all severities of asthma. Almost all the physicians "somewhat" or "strongly" believed that treating inflammation reduced the risk of bronchoconstriction (97%) and that ICS therapy was the "gold standard" therapy for asthma (95%) (Q2b).

**Figure 2:**
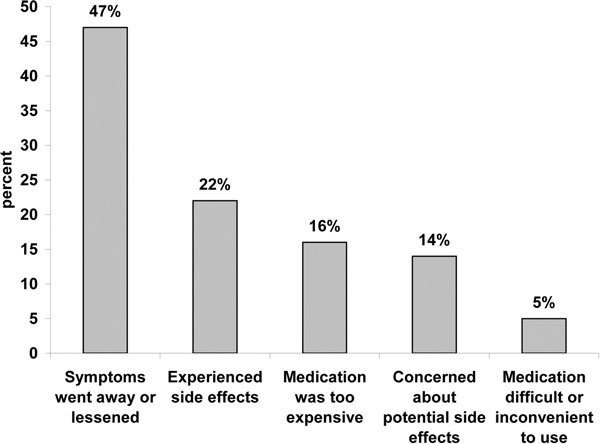
**Reasons for patients switching asthma medications**. Question: Since being diagnosed with asthma, have you ever switched from one asthma medication to another or discontinued an asthma medication because....?

With regard to their current asthma treatments, ~90% of patients were satisfied with the ease of use and effectiveness of the treatment, and ~85% were satisfied with the medication's time to onset, safety, and dosing frequency (Table 2; Q6a). Patients were least satisfied with the potential for side effects of asthma medications. The greatest reason for patients switching asthma medication was cessation or reduction of symptoms, followed by experience of side effects (Figure 2; Q7a).

**Figure 3:**
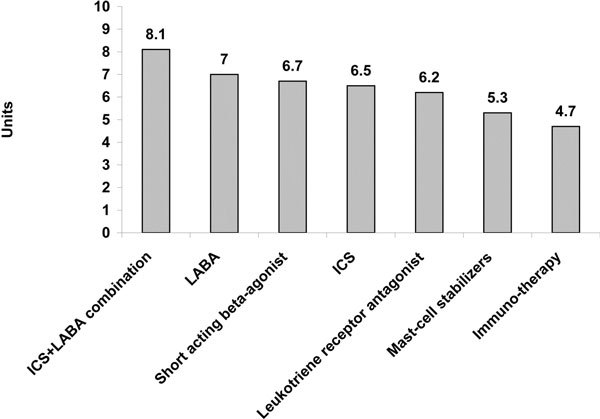
**Average physicians satisfaction with currently available asthma treatments**. Question: On a scale of 1-10 where "1" means "extremely dissatisfied" and "10" means "extremely satisfied" how satisfied are you with these currently available types of medications for treating asthma overall?

Of the currently available drug treatments for asthma, physicians were most satisfied with ICS and LABA combined therapy, and least satisfied with mast-cell stabilizers and immunotherapy (Figure 3; Q3b).

Most physicians in the survey reported asking patients if there was an allergic component to their asthma: 98% responded that they take a patient/family history of allergic symptoms and 99% asked about their asthma triggers (Q4b). Less than a third of the physicians tested for an allergic component via skin prick (18%) or assayed the patient's blood for specific allergen-IgE (32%) (Q4b). Most physicians (94%) reported that for patients with an allergic component to their asthma the physicians incorporate allergen avoidance into the asthma management plan, and 72% and 75% said they explained that the patient should remain on controller medication and referred the patient to an allergist or pulmonologist, respectively (Q5b). About half the patients in the survey with an allergic component to their asthma said that their doctor explained how to avoid allergic triggers (59%) and to use controller medication (50%) (Q8a). About a third of these same patients (34%) reported being referred to an allergist or other specialists (Q8a).

### Asthma Education

**Figure 4:**
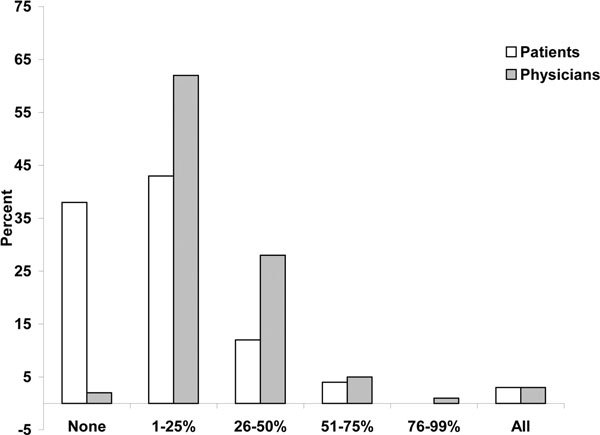
**Proportion of office visit devoted to asthma education**. Patient Question: During a typical visit with your doctor or health care professional, what percentage of the time do you or did you spend discussing how to improve techniques for successful management of your asthma? Physician Question: During a typical patient visit, what percent of time do you or other health professionals in your office spend on patient education regarding asthma?

Physicians and patients had differing views on the level and content of education regarding asthma and its treatments. Both patients and physicians responded that the treating physician is primarily responsible for patient education regarding asthma (86% and 95%, respectively) (Q9a and Q6b). Almost all the physicians (90%) in the survey responded that they spend up to 50% of the office visit discussing correct inhaler technique, development and execution of an individual management plan, and recognizing and monitoring symptoms (Figure 4; Q7b). Only 2% of physicians said they spent no time on asthma education. In contrast, 55% of patients reported that up to half the office visit was spent on asthma education and 38% said none of the visit was spent on educational issues (Figure 4; Q10a). Compared with physicians, patients reported less discussion of issues such as monitoring peak expiratory flow, proper inhaler technique, keeping daily symptom/medication diaries, disease management plan, and contacting patient support groups (Table 3; Q11a and Q8b). An indication that patient education may be inadequate was the finding that 40% of the patients responded that they did not know that asthma attacks can be fatal in patients with mild asthma (Q12a).

**Table 3:** Asthma Education Issues Patients Discuss with Their Health Care Providers

	Patients	Physicians
A plan for treating asthma	53%	87%
Correct inhaler technique	63%	95%
Keeping daily symptom/medication diaries	23%	50%
Monitoring peak expiratory flow	37%	84%
Contacting patient support groups	7%	26%

Patient question: Does your doctor or other healthcare professional in his or her office discuss any of the following with you?

Physician question: Do you regularly discuss the following with your asthma patients?

### Impact and Awareness of Medication Side Effects

**Figure 5:**
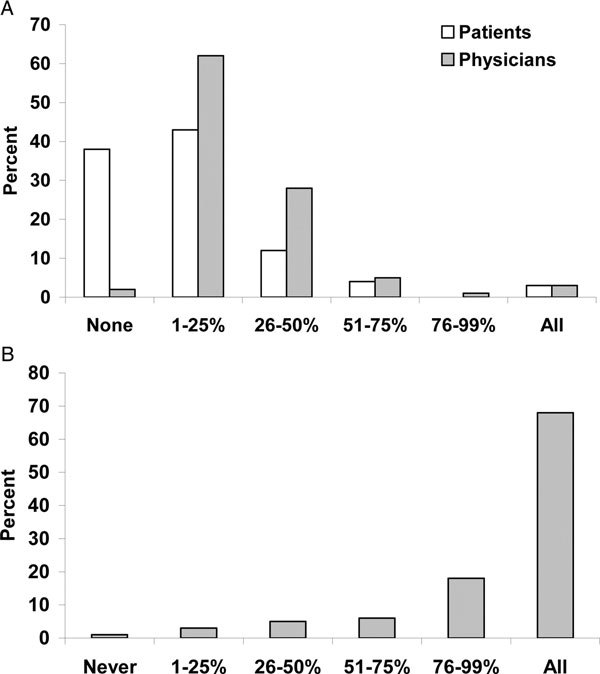
**(A) Frequency of discussions about short- and long-term side effects of asthma medications**. Physician questions: When you prescribe asthma medications to your patients, how often do you discuss local side effects such as oral thrush, pharyngitis or hoarseness? When you prescribe asthma medications to your patients, how often do you discuss the potential for systemic side effects such as osteoporosis, cataracts or glaucoma? Patient questions: How often do you or did you discuss short-term side effects of your asthma medication related to your mouth or throat--such as fungal infection, sore throat or hoarseness--with your doctor or health care professional? How often do you or did you discuss long-term side effects of your asthma medication--such as weight gain, weakening of the bones or changing bone density, cataracts or glaucoma--with your doctor or other health care professional? (B) Frequency of physicians informing patients of being prescribed an ICS. When you prescribe inhaled corticosteroids alone or in combination, on average, what percent of the time do you tell your patients that they will be taking a steroid?

Physician and patient perceptions of how frequently they discuss short-term and long-term side effects of asthma medication also differed. Both physicians and patients reported themselves as the person who initiated the discussion of side effects (85% for physicians and 62% for patients) (Q13a and Q9b). Furthermore, 90% and 77% of physicians reported sometimes or always discussing short-term and long-term side effects, respectively (Figure 5a; Q10b and Q11b). In contrast, 25% and 28% of patients said that their doctors sometimes or always discussed short- and long-term side effects, respectively, of their asthma medication (Figure 5a; Q14a and Q15a).

Many patients were unaware of the potential side-effects associated with ICS treatment (Q16a), which contrasted with physicians who answered that the majority of patients knew about potential side-effects (Table 4; Q12b). For example, while 39% of patients reported being unaware of long-term side effects, physicians believed that only 6% of patients were unaware. Similar discrepancies were also observed with short-term and cortisol/hypothalamic-pituitary-adrenal axis suppression (Table 4). Both patients and physicians were moderately concerned with ICS associated side effects (Table 4; Q17a and Q13b). Most physicians in this survey (68%) reported that they always informed a patient that they were being prescribed an ICS (Figure 5b; Q14b), and physicians perceived that patients were moderately concerned about taking a steroid (mean value was 5.1 on a 1-10 scale where "1" means "not at all concerned and "10" means "extremely concerned") (Q15b).

**Table 4:** Patient Awareness of Inhaled Corticosteroids Side Effects

	Patients		Physicians	
		
	Not Aware (%)*	Level of Concern*	Not Aware (%)†	Level of Concern†
Decreased cortisol production	46	3.9	14	4.4
Long-term side effects	39	5.1	6	5.1
Short-term side effects	19	4.6	5	5.7

*The following is a list of potential side effects of inhaled corticosteroids. On a scale of 1-10 where "1" mean "not at all concerned" and "10" means "extremely concerned," how concerned have you been with the following potential side effects, or were you not previously aware of these as potential side effects?

†On a scale of 1-10 where "1" means "not at all concerned" and "10" means "extremely concerned," how concerned are your patients with each of the following potential side effects of inhaled corticosteroids, or are they not aware of potential side effects?

Physicians and patients differed in their perception of how well patients recognize ICS-related side effects. Only 1% of patients interviewed said that they had experienced adrenal (cortisol) suppression, 8% said they had experienced long-term side effects, and 36% reported having short-term side effects (Q18a). In contrast, 17% of physicians reported that their patients had decreased cortisol production, 48% said their patients had long-term side effects, and 92% said their patients experienced short-term side effects (Q16b). Patients stated that side effects resulting from asthma medication caused them to consider switching (29%) or to switch medication (24%), consider skipping (26%) or skip doses (19%), or consider stopping (21%) or stop taking the medication entirely (22%), or change dosage (26%) (Q19a).

### Causes and Effects for Noncompliance

Perceptions of compliance differed between patients and physicians. When queried regarding the percentage of time they took their asthma medication according to their doctor or healthcare professional instructions, 35% of patients said they were fully compliant, 32% said they were compliant more than 50% of the time, and only 8% of patients said they were never compliant (Q20a). However, none of the physicians reported that their patients were fully compliant, 54% said that their patients were at least 50% compliant, and 5% said their patients were never compliant (Q17b). There are multiple reasons patients reported for being noncompliant; the most frequent responses were that symptoms went away, they felt they didn't need the medication that often, or they forgot to take the medication (Table 5; Q21a). These were also the most important reasons that physicians reported for lack of compliance by patients, but physicians also included lack of insurance coverage or medication expense (Table 5; Q18b). Reasons that physicians reported for knowing if their patients were complying with treatment regimen were: asking the patients (97%), knowing if their symptoms are controlled (89%), being told by the patient (88%), or they weighed the inhaler (5%) (Q19b).

**Table 5:** Reasons Patients Fail to Comply With Asthma Medication Treatment Regimen

	Patients (%)*	Physicians (%)†
Don't need to take if symptoms go away	5.4	7.9
Don't need to take it so often	5.2	7.5
Forget	5.2	7.3
Fear of steroids	4.1	5.6
Concerns about side effects	3.9	5.5
Concerns about becoming dependent	3.7	5.1
Inconvenience	3.7	6.6
Lack of insurance coverage or too expensive	3.6	7.2
Difficulty understanding the instructions	2.4	5.6

*Patient question: On a scale of 1-10 where "1" means "not at all important" and "10" means "extremely important," now important are the following reasons you don't or didn't always take your asthma medication as instructed?

†Physician question: On a scale of 1-10 where "1" means not at all important" and "10" means "extremely important," how important are the following reasons your patients don't take their asthma medication as instructed?

Physicians and patients differed in their perspectives of the consequences of non or partial adherence to treatment regimens. For example, 69% and 58% of patients reported increased symptoms and limited physical activity, respectively (Table 6; Q22a) as the primary consequence of none or partial adherence to treatment. Conversely, between 90% and 100% of physicians believed that their patients who were not 100% compliant experienced increased symptoms and limited physical activity (Table 6; Q20b). For each category, a higher percentage of physicians versus patients believed non or partial adherence had a detrimental effect, particularly regarding the frequency and severity of exacerbations, frequency of rescue medication use, nighttime awakenings, and increased physician visits and hospitalizations (Table 6).

**Table 6:** Patient and Physician Reported Consequence of Patients Not Taking Medication

	Patients (%)*	Physicians (%)†
Increase symptoms	69	100
Limited physical activity	58	91
Increased use of bronchodilator	46	99
Nighttime awakenings	39	93
More frequent asthma attacks or asthma exacerbations	35	100
More severe asthma attacks	27	94
More physician visits	25	99
More hospitalizations or ER visits	13	90
Absence from work	10	87
Life-threatening asthma attacks	10	65
Less interaction with friends and family	9	55

*Patient question: Have you ever experienced the following if you don't or didn't take your asthma medication as instructed?

†Physician question: Among your asthma patients, does non-compliance in their use of asthma medication cause...?

### New Medications

When questioned about the need for a new asthma medication, a similar percentage of patients (76%) and physicians (79%) thought that there was the need for a new treatment option in the US (Q23a and Q21b). When describing the required attributes for a new ICS, patients and physicians cited a lower potential for long-term side effects, efficacy equivalent to that of currently available ICS treatments and reduced propensity for oropharyngeal side effects (Q24a and Q22b). In addition, physicians thought the ability of the medication to be delivered without a spacer and that it have high lung deposition would be important treatment characteristics of a new ICS.

## Discussion

The GAPP survey was designed to identify differences in perspectives between physicians and patients on the treatment of asthma [12]. Overall, findings of the US subpopulation are consistent with the global survey results. Barriers to successful asthma treatment are created by notable differences between physician and patient perceptions regarding asthma and its effective treatment. The differences are particularly pronounced with regard to asthma education, awareness of side effects, disease symptoms, and adherence to asthma therapy.

The GINA [1] and NHLBI [8] guidelines recommend that physicians and patients collaborate and work as a team to ensure that patients are knowledgeable about the disease and have the skills to successfully self-manage their disease. Both the global [12] and US results indicate that in general, there is poor physician-patient partnership in treating the disease. The physician-patient partnership in the US may be less adequate than in other countries, as patients in the US reported that a lower proportion of time was spent on asthma education (16%) compared with the global survey patients (25%), and a greater proportion of US patients reported not knowing that an asthma attack could be fatal (40% vs 53%) (Table 7).

**Table 7:** Comparison of U.S. and Global GAPP Survey Results

	Patients		Physicians	
		
	US	Global	US	Global
Perception of asthma education issues				
Mean percent of office visit devoted to asthma education	16%	25%	28%	35%
Physician's that believe that they always discuss long-term side effects with patient	n.a.	n.a.	25%	26%
Physician's that believe that they always discuss short-term side effects with patient	n.a.	n.a.	42%	59%
Patient's who feel that the physician always discusses long-term side effects with them	4%	8%	n.a.	n.a.
Patient's who feel that the physician always discusses short-term side effects with them	6%	10%	n.a.	n.a.
Patient perception of asthma disease control				
Patients reporting to have mild-to-moderate asthma	94%	89%	n.a.	n.a.
Percentage of patients with unscheduled office visits or reported to ED	26%	38%	n.a.	n.a.
Patients not receiving medical attention for asthma	21%	11%	n.a.	n.a.
Patients that did not know that exacerbations in mild patients could be fatal	40%	53%	n.a.	n.a.
Perception of ICS side effects				
Patients not aware of long-term side effects	39%	31%	6%	7%
Patients not aware of short-term side effects	19%	20%	5%	3%
Patients experienced long-term side effects	8%	19%	48%	48%
Patients experienced short-term side effects	36%	34%	92%	93%
Perception of therapy adherence				
Percentage of time that patients are always adherent to asthma treatment regimen	35%	48%	0%	0%
Patients experienced increased symptoms when they did not take asthma medication	69%	69%	100%	99%

Similar to the global results,[12] US-specific findings indicate that there is a lack of asthma disease control (Table 7). Most US patients surveyed reported having mild-to-moderate asthma and that the disease had little influence on their daily activities. However, about 20% reported an unscheduled telephone call to the doctor, 20% reported an unscheduled office visit, and 6% either reported to the emergency room or were hospitalized for their asthma. Additionally, many patients underestimated their condition, because about 21% of the patients reported not receiving medical attention for their asthma and about 40% did not know that exacerbations in patients with mild asthma could be fatal. Similar observations regarding suboptimal asthma control have been noted in previous studies [14-16] and may reflect a lack of patient knowledge about their disease and the treatment outcomes they can expect.

Global [12] and US patients and physicians surveyed differed about their concern of ICS side effects (Table 7). Patients were more concerned about long-term side effects and physicians were more concerned about short-term side effects. Furthermore, only a few patients reported having long-term (8%) or short-term (36%) side effects while physicians believed most of their patients taking ICS or ICS/combination therapy had experienced long-term (48%) and short-term (98%) side effects. Compared with other countries in the survey, the US patients may be less cognizant of asthma medication side-effects: US patients versus the global survey patients were significantly less concerned about decreased cortisol production and long-term side effects (US results were 3.9 and 5.1 and global findings were 5.0 and 6.2, respectively, on a scale of 1-10 where "1" means not at all concerned and "10" means extremely concerned).

The global [12] and US findings indicate that compliance to therapy is poor (Table 7). Both the global and US physicians and patients reported that the most common reason for noncompliance was lack of symptoms. 'No symptoms, no disease' has previously been coined as a phrase when referring to patients' misunderstanding of their condition [17]. Self-medicating at a lower dose, forgetfulness, fear of steroids and concern over side-effects were also identified by physicians and patients as reasons for noncompliance. Such barriers to compliance have previously been observed and are believed to result from poor patient-physician communication regarding the disease and the long-term benefits of controller therapy [14,17-21]. In the US, physicians also reported insurance coverage and drug expense as other reasons for noncompliance.

Despite defining the reasons for poor compliance, patients and physicians surveyed both globally [12] and in the US observed that lack of compliance had serious consequences to health, quality of life, and resulted in potentially life-threatening exacerbations. Despite the fact that physicians and patients do recognize the link between therapy and disease, many patients still remain poorly controlled. Again, this may reflect inadequate disease education and lack of patient-physician communication. Treatment programs that actively involve both patients and physicians improve tangible outcomes for the patient (ie, decreased nighttime awakenings, increased physical activity, etc), increase the patient's understanding of the need to control their asthma effectively and the positive effects of controller medication and improves adherence [22-25].

Both the global [12] and US-specific results found that most physicians (both 95%) recognize ICS as the "gold standard," and believe that if you treat the inflammation you reduce the risk of bronchoconstriction. This is consistent with the findings that ICS therapy (either mono- or combined) was reported to be prescribed by the majority of physicians surveyed globally [12] and in the US (except for mild-intermittent asthma for which the majority of physicians prescribe short-acting *β*-agonists). In both groups of physicians LABA/ICS combination is the most common treatment (> 90%) for moderate-to-severe asthma as recommended by the guidelines [1,8]. Although both the global findings and US-specific results found that the majority of patients and physicians were satisfied with currently available asthma medication, they still cited the need for a new asthma medication. Major attributes cited for a new medication were lower potential for long-term side effects, similar efficacy as those presently available, and fewer side effects in the mouth and throat. Physicians also noted that a new ICS should have high lung deposition.

There are several aspects of this study that should be considered when interpreting the results. The range of asthma severity of the patients interviewed in this survey is not known. Asthma severity is known to influence the perceptions and behaviors of patients with regard to therapy, disease knowledge, and adherence [11,19]. The response rate for physicians (30%) is within the range that is typical for surveys performed by Harris Interactive, however, for patients the response was low (5%). To correct for selection bias associated with the physician and patient samples, the data for both the physician and patient were weighted so that the findings are likely representative of the general population. For each set of questions, the percent of patients responding in a specific way that were seeing a specialist, primary care physician, nurse, or not seeing anyone is not known. This may influence the interpretation of some results because in the global GAPP survey a patient's response appeared to be influenced in some cases by the type of doctor they were seeing for their asthma care [12]. In addition, it is important to note that the physician population in the US subgroup may have differed compared with the global population. A greater percentage of physicians participating in the global survey were pulmonologists (37%) compared with those participating in the US part of the survey (2%). In the US, the specialty most represented in the survey was internal medicine (50%).

Overall the US-specific findings of the GAPP survey are similar to those of the global [12] and of the Australian subpopulation [26] of the GAPP survey. Key findings from all 3 sets of data indicate that in general there is a lack of asthma control that likely results from poor adherence to therapy, and may at least in part reflect poor patient-physician partnership in treating this chronic disease. These findings are similar to another large worldwide survey that aimed at determining factors that influence asthma treatment [27].

The US findings may suggest a need for improved patient-physician partnership in the US and may support a change in the US from a more physician-led, technology-based, acute disease centered model of care to a more patient-centered medical home model. The patient-centered medical home model uses evidenced-based guidelines in treatment decisions and ensures adequate patient communication with healthcare professionals to help patients meet the challenges of a chronic disease such as asthma [22,28,29]. A central component of the model is to improve chronic disease care through a patient oriented intervention of an educational and supportive nature. This model stresses continuous relationships with the care team, individualized care according to the patient's needs and values, anticipation of the patient's needs, services based on evidenced based guidelines and cooperation among physicians [29]. The model also uses regular contact of the patient with the care team (through visits, email, phone calls, etc) to reinforce the patient's knowledge, self-management skills and confidence to become more responsible for and involved in their own disease management [29,30].

## End Note

Conflicts of Interest: Dr M. S. Blaiss: Dr Blaiss had received honoraria and been a consultant for the following companies: ALTANA Pharma, GlaxoSmithKline, Merck & Co, AstraZeneca and Schering Plough, Alcon, Teva, Novartis/Genentech, sanofi-aventis, and UCB; Dr M. A. Kaliner: Dr Kaliner has been involved in advisory boards and consultancy positions for the following companies: Alcon Laboratories, Glaxo-SmithKline, AstraZeneca, Greer Laboratories, Medimmune, MedPointe/Meda, Novartis-Genentech, Sanofi-Aventis, Schering Plough, Strategic Pharmaceutical Advisors and Teva Specialty Products/Ivax Laboratories. In addition to this, he has been involved in the speaker's bureaus for the following companies: Abbott Laboratories, Alcon Laboratories, Glaxo-SmithKline, MedPointe/Meda, Novartis-Genentech, Sanofi-Aventis, Schering Plough and Teva Specialty Products/Ivax Laboratories. Dr Kaliner has also received research grants from all asthma and allergy companies; Dr C. E. Baena-Cagnani: Dr Baena-Cagnani has been involved in the speaker's bureau for GlaxoSmithKline and sanofi aventis and has received speaker's honoraria from ALTANA, sanofi-aventis, UCD, AstraZeneca, FAES, Novartis, Schering-Plough, Lofarma (Italy), ALK-Abello and BioAllergy (Italy). Dr Baena-Cagnani has received clinical research support from Schering-Plough, sanofi-aventis, MSD, GSK, and Novartis.; Dr R. Dahl: Dr Dahl has been in advisory boards, participated in scientific clinical trials and been a speaker at meetings supported by ALK, Astra Zeneca, Boerhinger-Ingelheim Pfizer, GSK, UCB, Nycomed, MSD, Stallergen, TEVA, ONO, Dainippon, and Airsonett; Dr E. J. Valovirta: Dr Valovirta has been an advisory board member at ALK-Abello and MSD Finland, and has received honoraria and has been a consultant for the following companies: Altana Pharma, GSK, Merk & Co., Astra-Zeneca, Schering Plough, and ALK-Abello; Prof G. W. Canonica: Prof. Giorgio Walter Canonica has been: a scientific consultant as a single scientist or in national/international boards; a researcher in scientific trials in his university or in collaboration with other research institutions; and a speaker in scientific meetings, seminars and educational activities devoted to specialists, general practitioners and other healthcare professionals. These activities were totally or partially supported by the following commercial companies: A. Menarini, Alk-Abellò, Almirall, Altana-Nycomed, Anallergo, AstraZeneca, Boehringer Ingelheim, Chiesi Farmaceutici, Gentili, GSK, Lofarma, Merck Sharp & Dome, Novartis, Pfizer, Schering Plough, SigmaTau, Stallergenes, UCB Pharma, Uriach, and Valeas.

Supplemental digital content is available for this article. Direct URL citations appear in the text, and links to the digital files are provided in this article.
